# Phyletic patterns of bacterial growth temperature in *Pseudomonas* and *Paenibacillus* reveal gradual and sporadic evolution towards cold adaptation

**DOI:** 10.1093/ismeco/ycae163

**Published:** 2024-12-23

**Authors:** Kihyun Lee, Seong-Hyeon Kim, Seongjoon Moon, Sangha Kim, Changhan Lee

**Affiliations:** CJ Bioscience, Seoul 04527, Republic of Korea; Department of Biological Sciences, Ajou University, Suwon 16499, Republic of Korea; Department of Biological Sciences, Ajou University, Suwon 16499, Republic of Korea; Department of Biological Sciences, Ajou University, Suwon 16499, Republic of Korea; Department of Biological Sciences, Ajou University, Suwon 16499, Republic of Korea

**Keywords:** cold adaptation, psychrophile, psychrotroph, phylogenetics, *Pseudomonas*, *Paenibacillus*

## Abstract

Bacterial species adapt to cold environments with diverse molecular mechanisms enabling their growth under low ambient temperature. The emergence of cold-adapted species at macro-evolutionary scale, however, has not been systematically explored. In this study, we performed phylogenetic analysis on the growth temperature traits in the genera that occupy broad environmental and host niches and contain known cold-adapted species. Our results demonstrate that in the genus *Pseudomonas*, cold-adapted species formed a distinct and conserved clade, whereas in *Paenibacillus*, cold-adapted species were sporadically distributed throughout the phylogenetic tree. The cold-adapted clade of *Pseudomonas* exhibited genome-wide signatures of adaptation and possessed clade-specific genes. This indicates that there are diverse evolutionary patterns in the divergence of cold-adapted species among different bacterial genera.

Several molecular mechanisms in bacterial adaptation to low temperatures have been understood [1, 2], and the existence of cold-adapted species or clades of bacteria has been well described [3, 4]. However, surprisingly little is known about the tempo and mode of the evolution of cold-adapted species. For example, it remains unclear whether the cold-adaptation of bacteria tends to emerge independently in numerous lineages in a rapid tempo, or the trait tends to emerge gradually, forming a specialized branch of cold-adapting species. Here, we systematically analysed the evolutionary aspects of cold-adapted bacterial species within a genus.

Bacteria of the genus *Pseudomonas* inhabit diverse environments [5] and are widely present in cold environments such as those in Antarctica [6]. Therefore, we selected *Pseudomonas* as a model taxon for studying the evolution of cold adaptation. We collected and analysed the genomes, source information and growth environments of the type strains within this genus (Supplement Information).

We constructed a phylogenetic tree of *Pseudomonas* type strains and inspected the growth temperature data (Fig. 1A and Table S1). Interestingly, a clade within the *Pseudomonas* genus was identified comprised exclusively of cold-adapted species (19 out of 19 type strains in the clade). This clade has been labeled “L” for low-temperature adaptation. The L group included well-known cold-adapted bacteria such as *P. antarctica* PAMC 27494, *Pseudomonas* sp. Lz4W, and *P. psychrophila* RGCB 166 [7–9]. Alternatively, the cold-adapted clade can be defined more broadly to include species branching near the L clade (annotated as L’), in which case 38 out of 45 members of L + L’ are cold-adapted (Figs 1A and S1).

**Figure 1:**
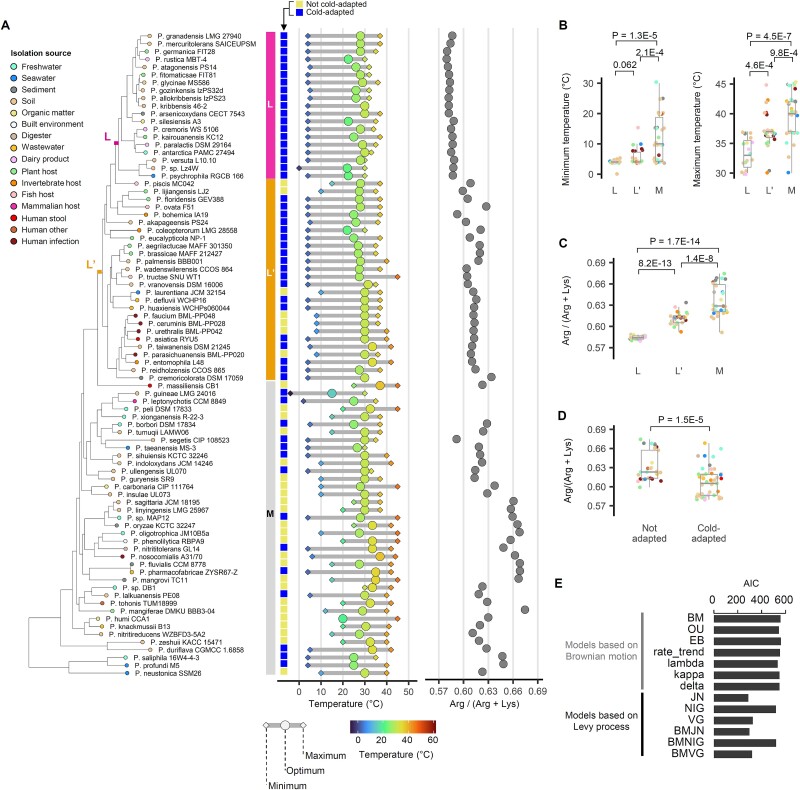
**Identification of a cold-adapted clade in *Pseudomonas*.** (A) Identification of the cold-adapted species clade (L or L’) on the phylogenetic tree of *Pseudomonas* species type strains based on single-copy core genes conserved among the genomes. The tree includes all *Pseudomonas* type strains for which the minimum, optimum, and maximum growth temperatures are available at BacDive. The isolation sources are indicated with color codes, and Arg/(Arg + Lys) ratios were displayed. (B) Comparison of the minimum and maximum growth temperatures between the L, L’ clade and the other species (M). (C) The mean Arg/(Arg + Lys) ratio of core proteins, averaged across all analyzed strains in the species. Boxplot compares L, L’ and M groups. (D) Arg/(Arg + Lys) ratio compared between the species that are cold-adapted versus not. Species whose type strain had minimum growth temperature of 5°C or below were defined as cold-adapted. (E) Ancestral state reconstruction modeling was conducted using various modes of trait evolution, fitting the minimum growth temperature data of the type strains to their phylogenetic trees (AIC: Akaike information criterion, BM: Brownian motion, incremental and free, OU: Ornstein-Uhlenbeck, incremental but stationary, EB: Early burst, rate_trend: Diffusion with linear trend through time, lambda: Pagel’s model transforming phylogeny, kappa: Pagel’s speciational model, delta:, JN: pure jump-normal process, NIG: pure normal inverse Gaussian process, VG: pure variance gamma process, BMJN: Jump-diffusion under jump-normal process, BMNIG: Jump-diffusion under negative inverse Gaussian process, BMVG: Jump-diffusion under variable gamma process).

Using our definition of L, L’ and M (i.e. all remaining taxa) groups, we observed a statistically significant difference in growth temperature among the L, L’, and M groups (Fig. 1B). In addition to the phenotypic trait of growth temperature, we analyzed the Arg/(Arg + Lys) ratio to further verify the clades as cold-adapted, as this ratio is a relevant marker for monitoring cold adaptation and decreases with cold adaptation [4]. We found that the Arg/(Arg + Lys) ratio in the L’ and L clades significantly reduced (Fig. 1A and C), and such clade-wise reduction was stronger than association with individual cold adaptation status (Fig. 1D). These results suggest that the L clades of *Pseudomonas* have undergone prolonged core genome-wide optimization and specialization for survival in low temperature that may limit their adaptability to high temperature. Notably, strains belonging to the L clades are predominantly environmental or plant isolates, suggesting that the adaptive evolution of these species is influenced by the temperature conditions of their habitats (Fig. 1 and S2, Tables S1 and S2).

To explore the mode of evolutionary processes that have driven cold-adaptations in the L clade of *Pseudomonas*, we fitted the extant type strains’ minimum growth temperature data to their phylogenetic trees using ancestral state reconstruction models assuming various modes of trait evolution (Fig. 1E). The pure jump Levy process model provided the best fit, closely followed by the jump-diffusion process, indicating that there were ancestral branches with accelerated shift in minimum growth temperature. In line with this observation, the patterns of protein family acquisitions throughout the *Pseudomonas* support the presence of clade-specific protein families in the cold-adapted clades (Fig. S3). Despite the limitations of pure Brownian diffusion model for this dataset, ancestral state reconstructed using such a model revealed ancestral divergences leading to L’ and L clades coupled with progressively lower minimum growth temperatures (Fig. S4).

We conducted similar analysis on the *Paenibacillus*, which are commonly found in various environments, including cold conditions (Fig. 2A and Fig. S5) [10]. Remarkably, in contrast to the previously analyzed *Pseudomonas*, the cold-adapted species tend not to form a distinct clade. Instead, they are sporadically and widely distributed across the lower branches of the phylogenetic tree. To quantitatively assess such differences between the two genera, we calculated correlation between minimum growth temperature and phylogeny. Based on Frists Purvis D tests in *Pseudomonas*, a negative D value (*D* = −0.17, *P* = 0.001) suggests strong phylogenetic clustering, indicating that cold-adapted clades likely emerged in ancestral clades and has been retained with minimal changes. In contrast, *Paenibacillus* shows a D value closer to randomness (*D* = 0.80, *P* = 0.15), implying a more dispersed pattern of cold adaptation, likely driven by environmental pressures rather than conserved inheritance. Simpler correlation tests between the phylogenetic distance and the difference in minimum growth temperature yielded similar results (Table S3). Analysis of the core genome mean Arg/(Arg + Lys) ratio also did not reveal characteristic skew in the genomes of cold-adapted type strains (Figs 2B and S5). With *Paenibacillus* dataset, the core genome-wide mean Arg/(Arg + Lys) ratio was not significantly different between cold-adapted and the other species (Fig. 2B) and none of the tested ancestral reconstruction models showed substantially better fit with the data (Fig. 2C), possibly indicating lack of prolonged cold adaptation required for genome-wide optimization and poor correlation between evolutionary history and the minimum growth temperature traits, respectively. These results demonstrate a difference in the evolutionary speciation pattern for cold-adapted species in *Paenibacillus* compared to *Pseudomonas*.

**Figure 2:**
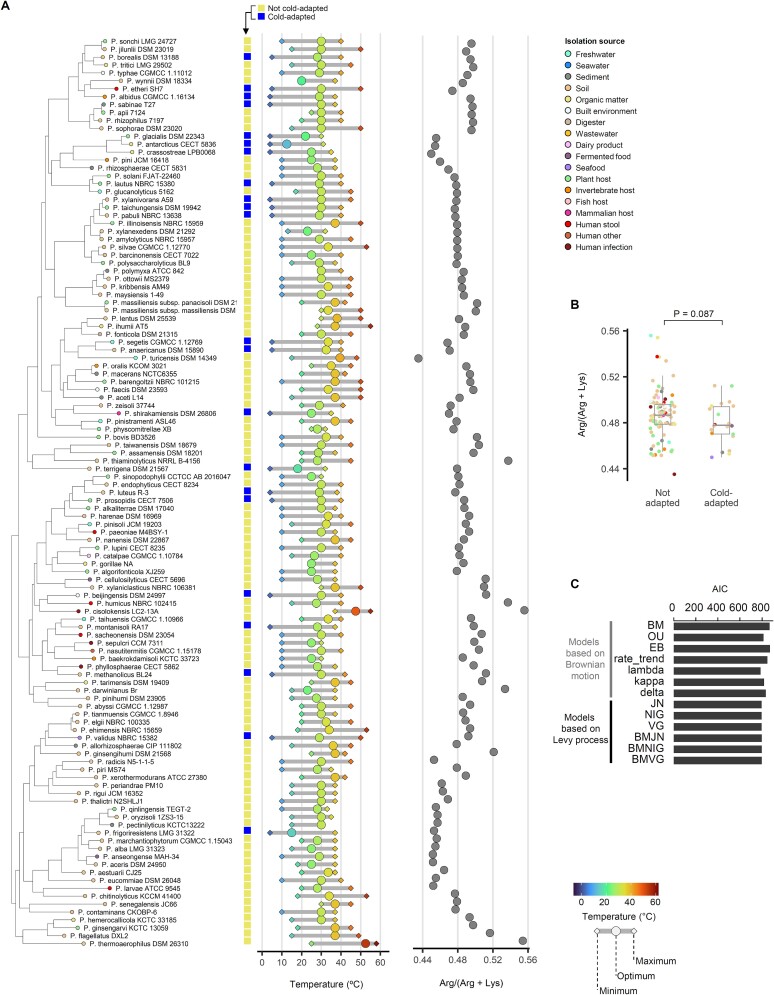
**Sporadic distribution of cold-adapted species in *Paenibacillus***. (A) Distribution of growth temperature ranges of the type strains in *Paenibacillus* across the phylogenetic tree of the strains based on single-copy core genes. The tree includes all *Paenibacillus* type strains for which the minimum, optimum, and maximum growth temperatures are available at BacDive. The isolation sources are indicated with color codes, and core genome-wide mean Arg/(Arg + Lys) ratios were displayed. (B) the mean Arg/(Arg + Lys) ratio of core proteins, averaged across all analyzed strains in the species. Boxplot compares the species that are cold-adapted versus not. Species whose type strain had minimum growth temperature of 5°C or below were defined as cold-adapted. (C) Fitness of ancestral state reconstruction models with the minimum growth temperature data of the type strains and their phylogenetic trees.

Collectively, these results suggest two distinct patterns of cold-adapted speciation across different genera. One pattern, as observed in *Pseudomonas*, involves punctuated evolution that forms ancestral cold-adapted lineage leading to diversification of cold-adapted species within the clade, accompanying long-term genome-wide optimization. The other pattern, exemplified by *Paenibacillus*, is episodic speciation from diverse non-cold-adapted ancestors. These findings imply that cold adaptation can occur in different evolutionary contexts. This might be due to the emergence of predominant ancestral species. Alternatively, mechanisms for cold adaptation could occur in two ways: swiftly through the acquisition or loss of genes, or gradually over a long period through incremental changes across the entire genome.

## Supplementary Material

low_temperature_species_manuscript_supplementary_CL17_ycae163

supplementary_table1_CL5_ycae163

supplementary_table2_CL1_ycae163

## Data Availability

All data generated or analyzed during this study are included in this published article and its supplementary information files.
