# Supplementary material for: Phyletic patterns of bacterial growth temperature in Pseudomonas and Paenibacillus reveal gradual and sporadic evolution towards cold adaptation
Source: ISME Commun. 2024 Dec 23;4(1):ycae163. doi: 10.1093/ismeco/ycae163 (PMC11694702; doi:10.1093/ismeco/ycae163)
Supplement: low_temperature_species_manuscript_supplementary_CL17_ycae163 [file low_temperature_species_manuscript_supplementary_cl17_ycae163.pdf]

Supplementary materials

Phyletic Patterns of Bacterial Growth Temperature in *Pseudomonas* and *Paenibacillus* Reveal Gradual and Sporadic Evolution Towards Cold Adaptation

Kihyun Lee[2#], Seong-Hyeon Kim[1*], Seongjoon Moon[1*], Sangha Kim[1*], Changhan Lee[1#]

[1] Department of Biological Sciences, Ajou University, Suwon 16499, Republic of Korea

[2] CJ Bioscience, Seoul 04527, Republic of Korea

[*] equally contributed as second authors

[#] Correspondence

List of supplementary materials:

- Materials and methods
- Supplementary figures
    - Fig. S1: Phylogenetic tree of *Pseudomonas* genus
    - Fig. S2: Strain isolation source distribution of the *Pseudomonas* species
    - Fig. S3: Genomic signatures of cold adapted clades in *Pseudomonas*
    - Fig. S4: Growth temperatures inferred through ancestral state reconstruction
    - Fig. S5: Genomic signatures of cold adapted clades in *Paenibacillus*
- Supplementary table
    - Table S1: List of type strains and dataset analyzed in this study (provided as a separate excel file)
    - Table S2: List of non-type strains and dataset analyzed in this study (provided as a separate excel file)
    - Table S3: Kendall's Tau and Spearman's Rho analyses

Materials and methods

Data collection

We collected the list of type strains and their growth temperature information including growth temperature ranges and optimal growth temperatures, from the BacDive database [1]. We focused on four genera, *Pseudomonas* and *Paniebacillus*, as these genera contained multiple psychrophilic and mesophilic strains with available genome sequence accession numbers. The temperature information obtained from the BacDive was verified through reference check to confirm whether these values were experimentally proven. By doing so, we corrected any discrepancies and cited the relevant references in supplementary table 1. Species without minimum or maximum growth temperatures were excluded from the main phylogenetic analyses. Since terminologies for cold-adapted bacteria are not clearly resolved yet [2], we classified the thermal niche of the type strains based on their reported minimum growth temperatures, categorizing species as cold-adapted if their minimum growth temperature was not higher than 5°C [3]. The GenBank genome assembly accession numbers of the strains were collected and used in the phylogenetic analyses. The list of type and non-type strains analyzed in this study, along with their genome sequence accession numbers, basic source information, and the growth temperature metadata, are provided in the supplementary table 1 and 2.

There are some limitations of the collected data. The full range of growth temperatures may not have been tested for the strains. Additionally, variations in growth conditions, such as the type of medium used, could impact growth, leading to inaccuracies in the temperature data. Differences in sampling across genera could also present limitations, as variation in sample size and differences in phylogenetic tree shapes, such as branching depth, may influence interpretations of cold adaptation. The tree structures in our study are relatively moderate and not extreme, i.e., from the visual inspection of the trees in Fig. 1 and Fig. 2, the trees are neither dominated by deep branches (star-like) or dominated by shallow branches. Nonetheless, it is not possible to completely exclude the possibility that sampling discrepancies had an effect on the observed difference in evolutionary patterns. Additionally, differences in the evolutionary age of genera may lead to notable differences in whether cold-adapted strains are clustered or more broadly distributed. Additionally, it is worth noting that the species in each of the genera analyzed in this study span multiple genera according to the genome-based taxonomic classification by GTDB (https://gtdb.ecogenomic.org/tree?r=f__Pseudomonadaceae and https://gtdb.ecogenomic.org/tree?r=g__Paenibacillus). Precisely, the *Paenibacillus* species included in this study belong to Paenibacillus (n = 32), Paenibacillus_A (n = 15), Paenibacillus_E (n = 7), Paenibacillus_Z (n = 7), Paenibacillus_G (n = 6), Paenibacillus_S (n = 4), Paenibacillus_B (n = 3),

Paenibacillus_D (n = 2), Paenibacillus_O (n = 2), Paenibacillus_T (n = 2), Paenibacillus_I (n = 2), Paenibacillus_J (n = 1), Paenibacillus_F (n = 1), Paenibacillus_V (n = 1), Paenibacillus_H (n = 1), Paenibacillus_L (n = 1), Paenibacillus_K (n = 1), Paenibacillus_W (n = 1) under GTDB system. In cases of the *Pseudomonas* species included in this study belong to Pseudomonas_E (n = 59), Pseudomonas_K (n = 3), Pseudomonas (n = 2), Pseudomonas_F (n = 2), Pseudomonas_B (n = 1), Pseudomonas_T (n = 1) under the GTDB classification. Since GTDB enforce the taxa at a given rank to be defined at homogenous phylogenetic depth, the fact that *Paenibacillus* species analyzed in this study spread across a larger number of GTDB-defined genera might mean that the phylogenetic diversity represented in the analyzed *Paenibacillus* dataset might be greater than that of *Pseudomonas* analyzed in this study. It is possible that difference in the scale of phylogenetic diversity captured in each genus' dataset has effect on the degree of clustering of cold-adapted species.

Phylogenetic analyses

We reconstructed two types of phylogenetic trees in this study. The trees containing all strains in a genus (Fig. S1) were reconstructed based on the pre-defined BUSCO markers for the corresponding order-level taxon. The smaller trees, with fully available growth temperature range data (Fig. 1, Fig. 2) were reconstructed based on the single-copy core genes of each genus that we determined in this study. We selected an outgroup from a closely related neighboring genus to establish the root position (for *Pseudomonas*, using *Cellvibrio japonicus* Ueda107 genome GCA_000019225.1 and *Azomonas agilis* DSM 375 genome GCA_007830255.1; for *Paenibacillus*, using *Brevibacillus laterosporus* DSM 25 genome GCA_002706795.1 and *Brevibacillus brevis* NCTC2611 genome GCA_900637055.1).

For BUSCO-based phylogenetic analyses, we used BUSCO 5.7.1 to extract the sequences belonging to the predefined phylogenetic marker orthologs from each input genomes [4]. We used lineage data "pseudomonadales_odb10" for the analyses of *Pseudomonas* strains. The BUSCO software conveniently produced sequence fasta files per orthologous family.

To define the single-copy core genes of each genus, we first defined the protein-coding regions in the studied genomes using Prokka 1.14.6 [5]. Next, we determined the clusters of similar proteins using Diamond cluster tool, with 50% identity and 80% coverage thresholds [6]. Protein clusters that contained one protein per every input strain (e.g., 100% frequency, without any duplication) were defined as the core single-copy genes of the genus and the sequences belonging to those clusters were collected.

To generate multiple sequence alignments at each locus, we used super5 algorithm in Muscle 5.1 to create alignments at protein sequence level [7], and created codon-by-codon alignments from the protein alignments and nucleotide sequences using PAL2NAL v14 [8]. The aligned sequences

generated per ortholog were later concatenated into a single super-matrix.

We used IQ-Tree 2.0.7 to reconstruct maximum-likelihood phylogenetic trees from the concatenated alignments [9]. For BUSCO marker trees, we applied “-m GTR+F” substitution model across datasets from all genera. For core genome trees, we used the best-fit model determined by IQ-Tree’s ModelFinder for each dataset:“SYM+R10” for *Paenibacillus*, “GTR+F+R9” for *Pseudomonas*.

We derived chronogram (i.e., branch lengths correspond to relative time span rather than substitutions) from the original phylogenetic tree using chronos function implemented in the ape package [10]. Note that date calibration was not performed since it is not feasible to calibrate internal node dates of our trees based on the given scale of variation in sampling dates among almost contemporary collection of type strains. The chronogram was used in Fig. S3. We performed ancestral state reconstruction to infer the minimum and maximum growth temperature at internal nodes based on the chronogram and the temperature data of extant type strains. To test how well various trait evolutionary models fit with the minimum growth temperature data and the phylogenetic trees of *Pseudomonas* and *Paenibacillus,* we evaluated Akaike information criteria (AIC) estimated from application of various models: "BM", "OU", "EB", "rate_trend", "lambda", "kappa", and "delta" models as provided with fitContinuous function in geiger package [11], and Levy process-involving “JN”, “VG”, “NIG”, “BMJN”, “BMVG”, “BMNIG” models as provided with fit_reml_levy function in pulsR package [12]. Ancestral reconstruction result shown in Fig. S3 was inferred with Brownian motion-based method implemented in fastAnc function of Phytools package [13]. Phylogenetic figures were visualized with ggtree package [14].

Although the L clades appeared intuitively in the phylogenetic tree of *Pseudomonas*, several factors were considered in defining them. Notably, the type strains within the L clades are mostly isolated from environmental sources. In contrast, the species located just below the L' clade, on a separate branch, is associated with a mammalian host (Fig. 1A, S2). Additionally, ancestral state reconstruction analysis reveals distinct branch differentiation for the L clades, suggesting a clear boundary for their definition (Fig. S4).

Statistical analyses

To assess differences in growth temperature between clades, non-parametric Wilcoxon rank-sum test was used. To assess the correlation between the cophenetic distance on the trees and the difference in growth temperature (i.e., to test if the species pairs close on the tree have smaller difference in growth temperature) we used Spearman’s and Kendall’s correlation tests using R core functions in R 4.3.2. We also used a more phylogenetics-framed test for clustering of growth temperature distribution on the phylogenetic trees of *Pseudomonas* and *Paenibacillus* type strains using Fritz and Purvis' D statistic

implemented in phylo.d function of caper package [15].

References

1. Reimer LC, Sardà Carbasse J, Koblitz J, Ebeling C, Podstawka A, Overmann J. Bac Dive in 2022: the knowledge base for standardized bacterial and archaeal data. *Nucleic Acids Research* 2022; **50**: D741–D746.
2. Cavicchioli R. On the concept of a psychrophile. *The ISME Journal* 2016; **10**: 793–795.
3. De Maayer P, Anderson D, Cary C, Cowan DA. Some like it cold: understanding the survival strategies of psychrophiles. *EMBO reports* 2014; **15**: 508–517.
4. Manni M, Berkeley MR, Seppey M, Simão FA, Zdobnov EM. BUSCO update: novel and streamlined workflows along with broader and deeper phylogenetic coverage for scoring of eukaryotic, prokaryotic, and viral genomes. *Molecular biology and evolution* 2021; **38**: 4647–4654.
5. Seemann T. Prokka: rapid prokaryotic genome annotation. *Bioinformatics* 2014; **30**: 2068–2069.
6. Buchfink B, Ashkenazy H, Reuter K, Kennedy JA, Drost H-G. Sensitive clustering of protein sequences at tree-of-life scale using DIAMOND DeepClust. *bioRxiv* 2023; 2023–01.
7. Edgar RC. Muscle5: High-accuracy alignment ensembles enable unbiased assessments of sequence homology and phylogeny. *Nature Communications* 2022; **13**: 6968.
8. Suyama M, Torrents D, Bork P. PAL2NAL: robust conversion of protein sequence alignments into the corresponding codon alignments. *Nucleic acids research* 2006; **34**: W609–W612.
9. Minh BQ, Schmidt HA, Chernomor O, Schrempf D, Woodhams MD, Von Haeseler A, et al. IQ-TREE 2: new models and efficient methods for phylogenetic inference in the genomic era. *Molecular biology and evolution* 2020; **37**: 1530–1534.
10. Paradis E. Molecular dating of phylogenies by likelihood methods: a comparison of models and a new information criterion. *Molecular phylogenetics and evolution* 2013; **67**: 436–444.
11. Pennell MW, Eastman JM, Slater GJ, Brown JW, Uyeda JC, FitzJohn RG, et al. geiger v2. 0: an

expanded suite of methods for fitting macroevolutionary models to phylogenetic trees. *Bioinformatics* 2014; **30**: 2216–2218.

12. Landis MJ, Schraiber JG. Pulsed evolution shaped modern vertebrate body sizes. *Proceedings of the National Academy of Sciences* 2017; **114**: 13224–13229.
13. Revell LJ. phytools 2.0: an updated R ecosystem for phylogenetic comparative methods (and other things). *PeerJ* 2024; **12**: e16505.
14. Yu G, Smith DK, Zhu H, Guan Y, Lam TT. ggtree: an R package for visualization and annotation of phylogenetic trees with their covariates and other associated data. *Methods in Ecology and Evolution* 2017; **8**: 28–36.
15. Fritz SA, Purvis A. Selectivity in mammalian extinction risk and threat types: a new measure of phylogenetic signal strength in binary traits. *Conservation Biology* 2010; **24**: 1042–1051.

Supplementary figures

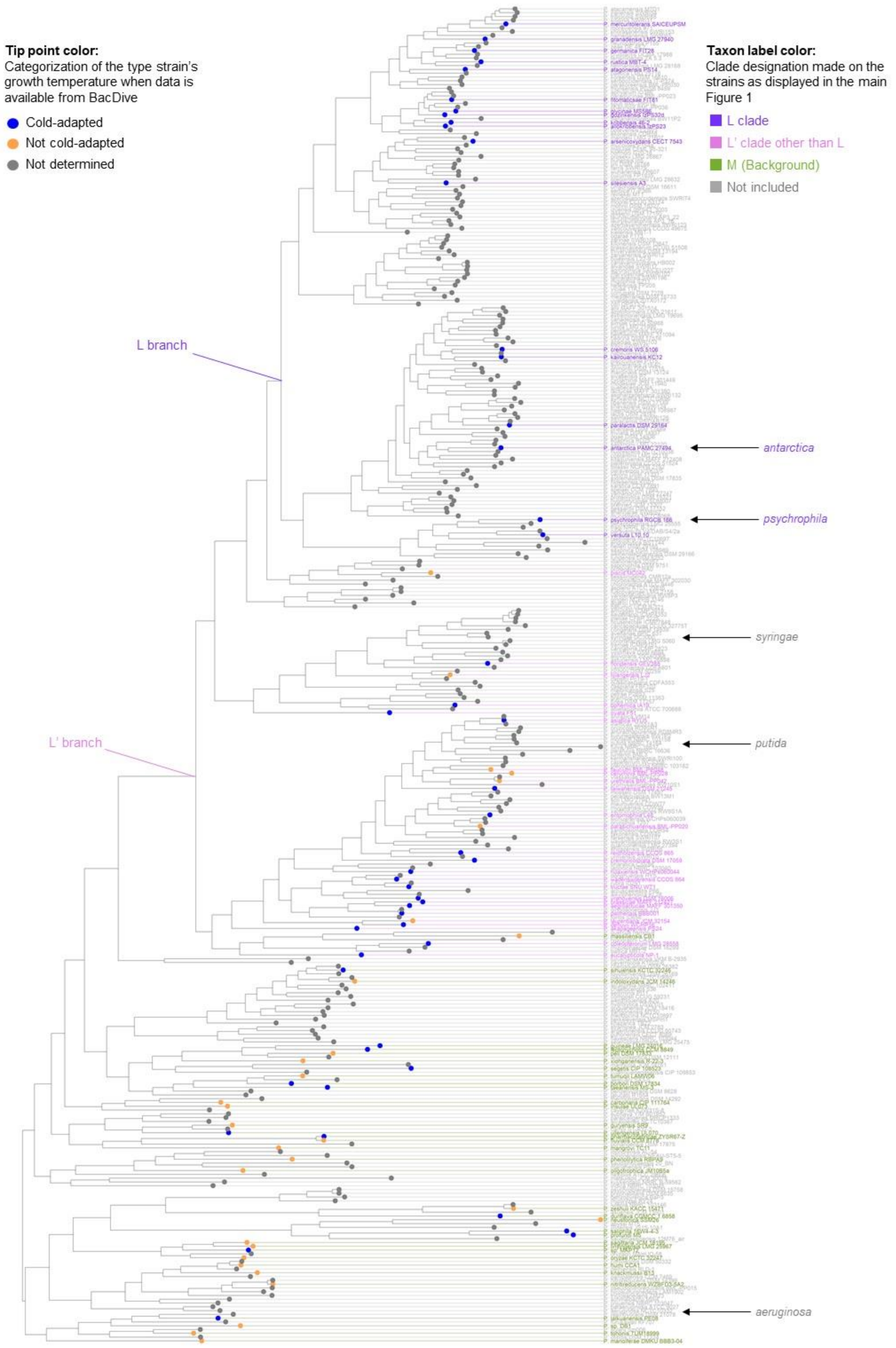

Fig. S1: Phylogenetic tree of *Pseudomonas* genus.

The tree includes all type strains of *Pseudomonas* species that have a genome assembly available in GenBank. The tree was reconstructed from the BUSCO marker set pseudomonadales_odb10. The strains used in Fig. 1, i.e., which have minimum, optimum, and maximum growth temperature data, are marked with color-coded taxon names and color-coded tip points. The taxon label colors are given according to the clade designation shown in Fig. 1. The tip point colors follow the categorization of strain's temperature range into L, L' and M groups. The most recent common ancestor branches for L and L' clades are depicted with text labels. Well known cold-adapted species such as *P. antarctica* and *P. psychrophila*, along with the well-studied species such as *P. syringae*, *P. putida*, and *P. aeruginosa*, are marked with arrows.

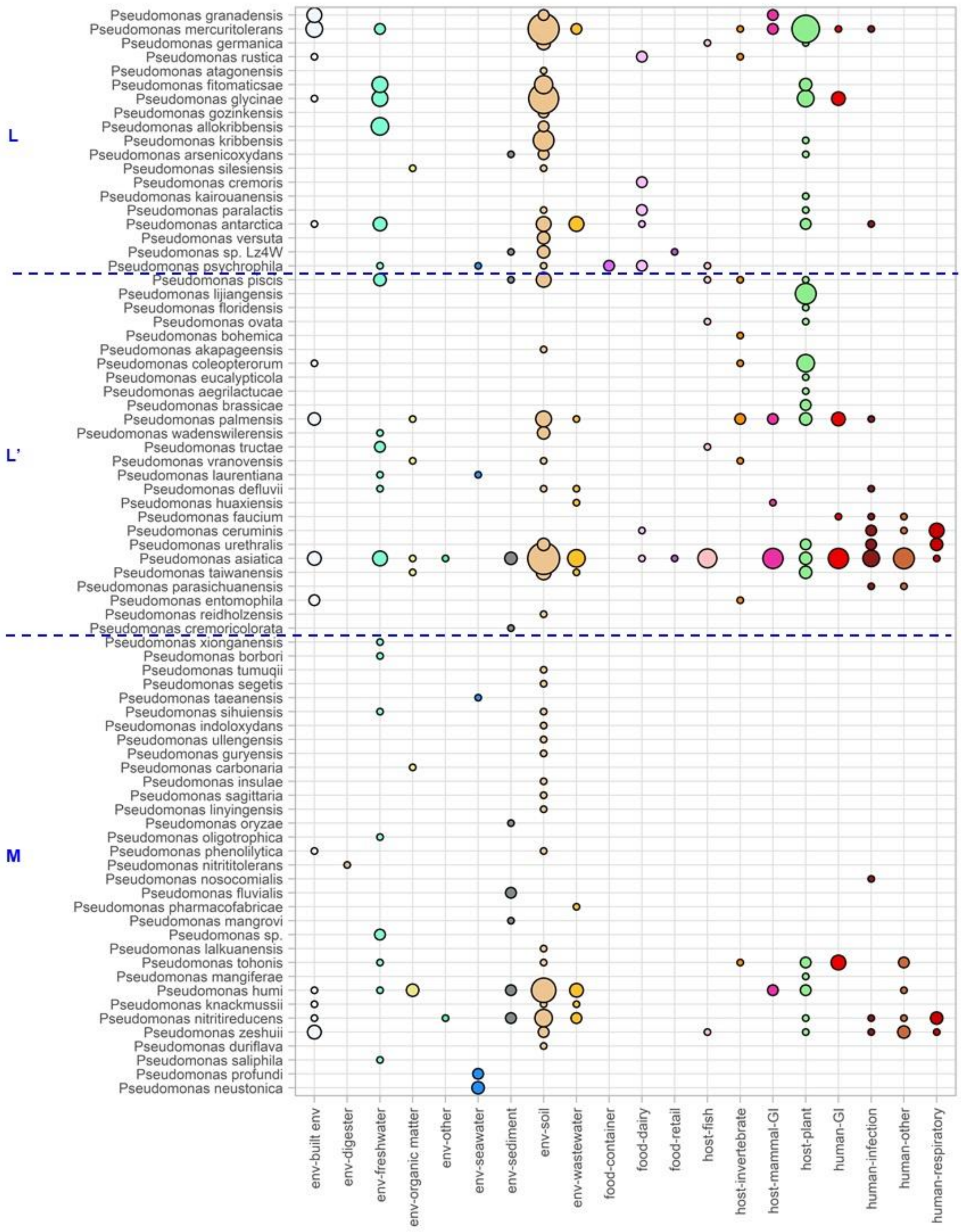

Fig. S2: Strain isolation source distribution of the *Pseudomonas* species

Bubble size correlates with the number of strains. Color of the bubbles reflect the source category labelled on the x axis. Species are ordered according to the order appearing in the phylogenetic analysis.

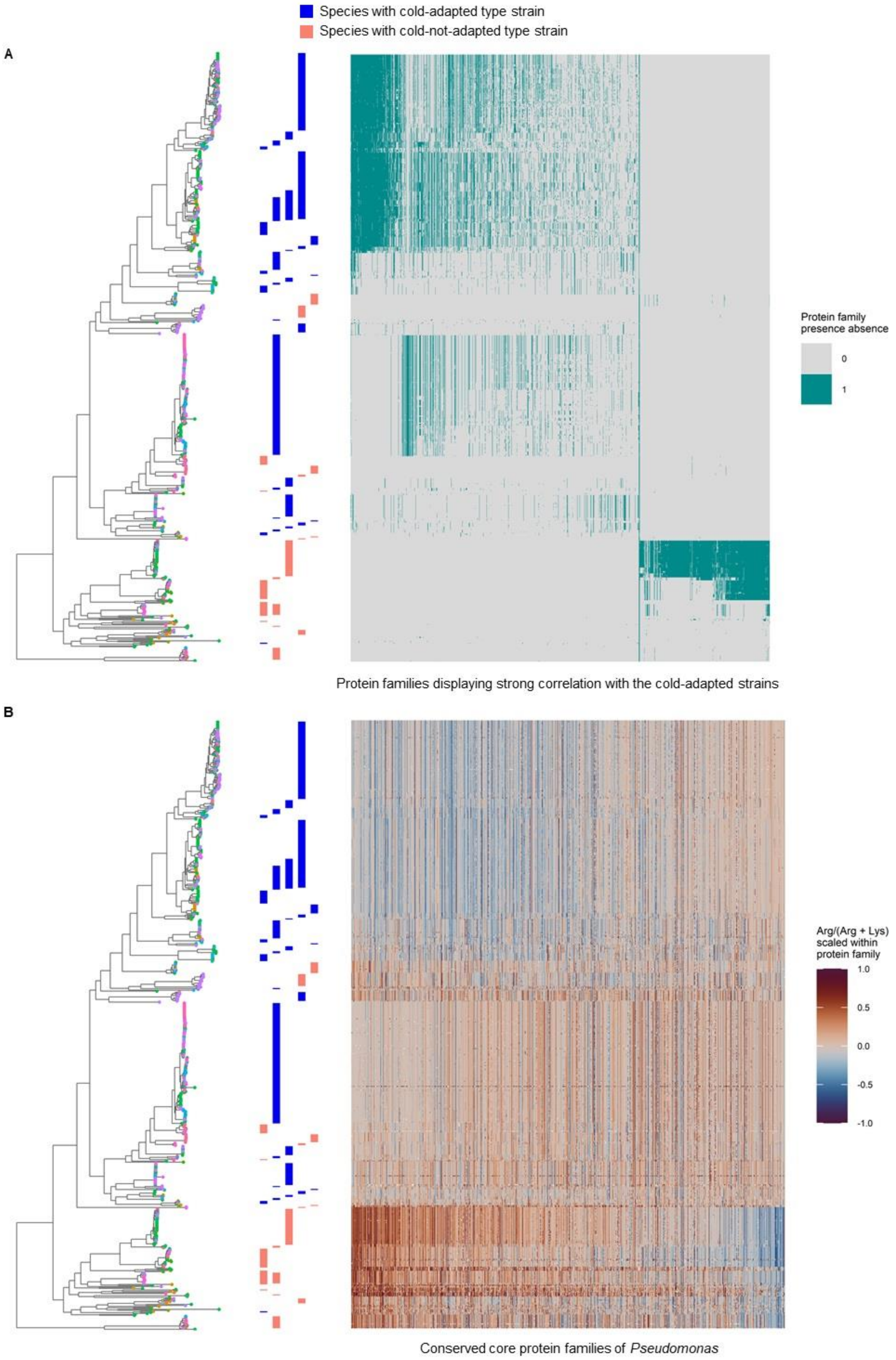
Species with cold-adapted type strain
Species with cold-not-adapted type strain
A
Protein family presence absence
0
1
Protein families displaying strong correlation with the cold-adapted strains
B
Arg/(Arg + Lys) scaled within protein family
1.0
0.5
0.0
-0.5
-1.0
Conserved core protein families of *Pseudomonas*

Fig S3: Genomic signatures of cold adapted clades in *Pseudomonas*

(A) Heat map visualization of the presence or absence patterns of the protein families in association with the cold adapted species, on the phylogenetic tree of *Pseudomonas* type strains. Protein families with odd ratio of $> 1,000$ or < 0.001 are shown, and the protein families are ordered according to the odd ratio. The odd ratios were calculated from logistic regression between the protein family presence/absence and the species type strain's cold-adaptation status. (B) Heat map visualization of core genome protein sequences' Arg/(Arg + Lys) ratio in association cold adapted species. Protein families are ordered along the x-axis according to the point-biserial correlation coefficient between the Arg/(Arg + Lys) ratio and the type strain's cold-adaptation status (0, minimum growth temperature $>$ 5, not cold-adapted; 1, minimum growth temperature < 5, cold-adapted)

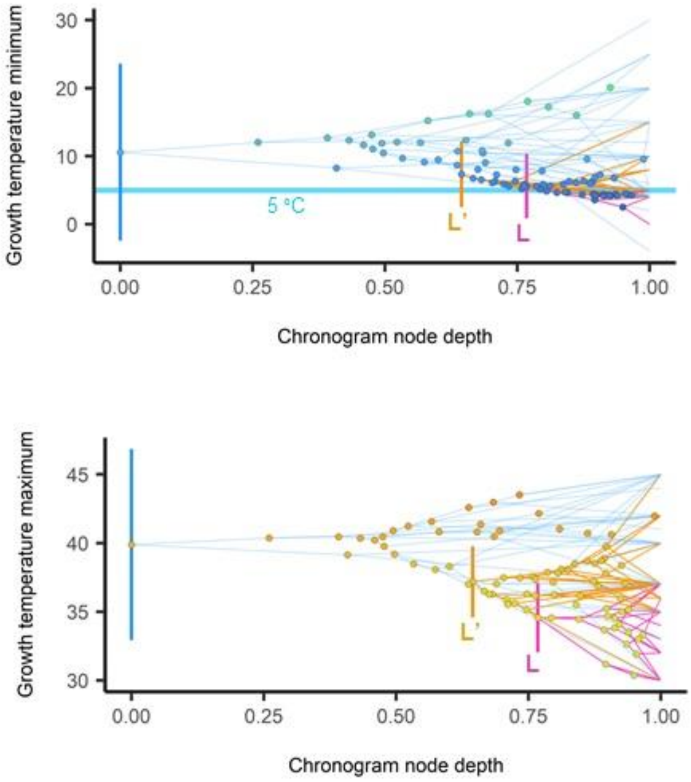

Fig. S4: Growth temperatures of *Pseudomonas* inferred through ancestral state reconstruction

Growth temperatures (minimum growth temperature: upper panel, maximum growth temperature panel: lower panel) inferred through ancestral state reconstruction at each internal node are shown for each internal node. X-axis coordinates correspond to node depth in the chronogram. Vertical ranges placed at the ancestral nodes corresponding to the most recent common ancestors of the genus, the L' clade, and the L clade display the 95% confidence interval of the growth temperatures inferred for these nodes. Branches inside L or L' clades are colored differently from the other branches.

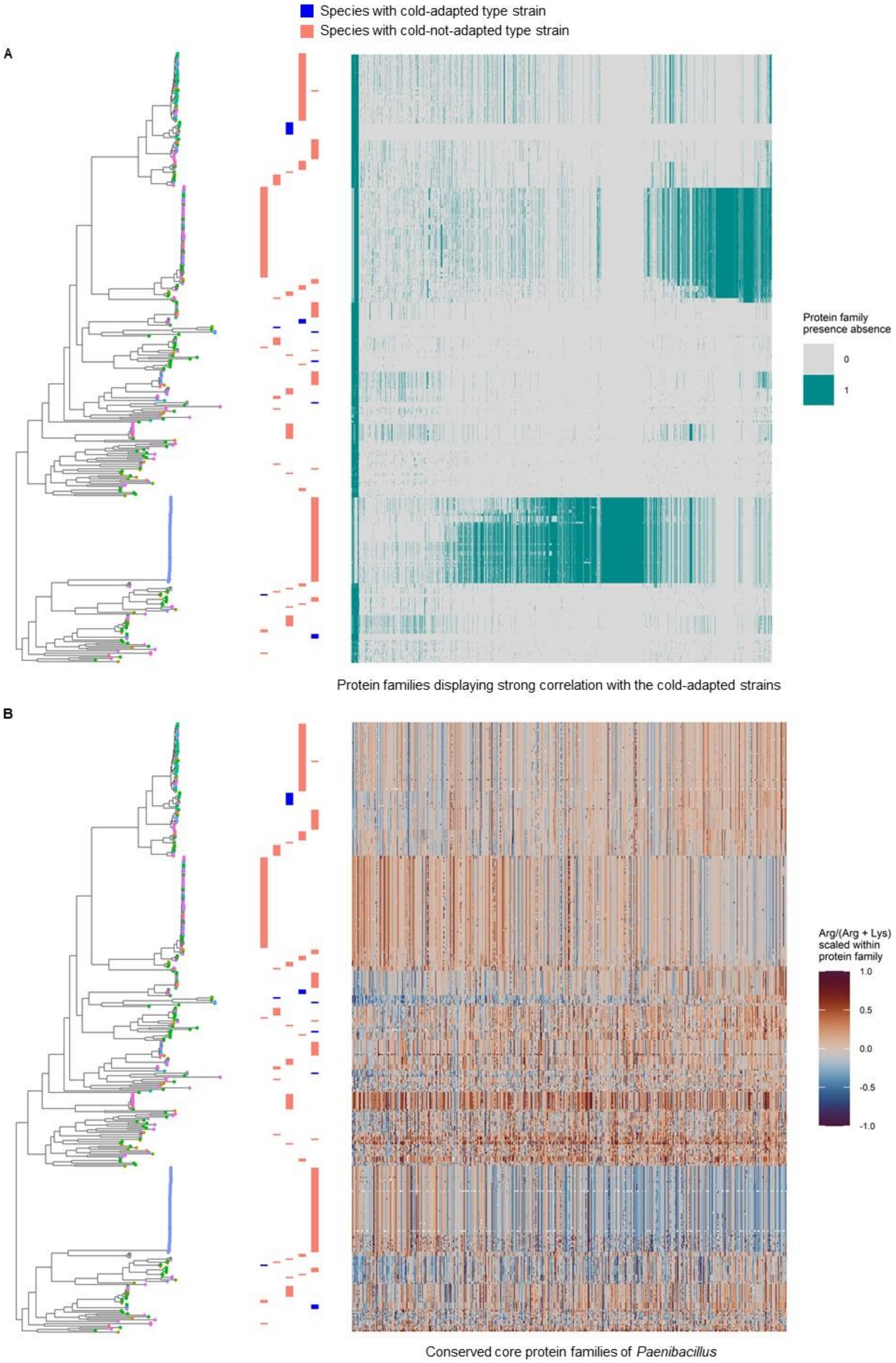
Species with cold-adapted type strain
Species with cold-not-adapted type strain
A
Protein family presence absence
0
1
Protein families displaying strong correlation with the cold-adapted strains
B
Arg/(Arg + Lys) scaled within protein family
1.0
0.5
0.0
-0.5
-1.0
Conserved core protein families of *Paenibacillus*

210

Fig S5: Genomic signatures of cold adapted clades in *Paenibacillus*

(A) Heat map visualization of the presence or absence patterns of the protein families in association with the cold adapted species, on the phylogenetic tree of *Paenibacillus* type strains. Protein families with odd ratio of > 1,000 or < 0.001 are shown, and the protein families are ordered according to the odd ratio. The odd ratios were calculated from logistic regression between the protein family presence/absence and the species type strain's cold-adaptation status. (B) Heat map visualization of core genome protein sequences' Arg/(Arg + Lys) ratio in association cold adapted species. Protein families are ordered along the x-axis according to the point-biserial correlation coefficient between the Arg/(Arg + Lys) ratio and the type strain's cold-adaptation status (0, minimum growth temperature > 5, not cold-adapted; 1, minimum growth temperature < 5, cold-adapted)

Tables

Table S1: List of type strains and dataset analyzed in this study

- provided as a separate excel file

-Table S2: List of non-type strains and dataset analyzed in this study

- provided as a separate excel file)

Table S3: Kendall's Tau and Spearman's Rho analyses

Genus	Kendall’s Tau (τ)	p-value for τ	Spearman's Rho (ρ)	p-value for ρ
Pseudomonas[1]	0.196	2.3×10^{-59}	0.273	2.8×10^{-59}
Paenibacillus[2]	0.008	0.37	0.011	0.36

[1] Both coefficients indicate a significant positive correlation, with Spearman's showing a slightly stronger relationship. This suggests a correlation between growth temperature and phylogeny based on genomic data in *Pseudomonas* species

[2] These results demonstrate no significant correlation between growth temperature and phylogeny in *Paenibacillus*. This indicates a difference in the evolutionary speciation pattern for cold-adapted species in *Paenibacillus* compared to *Pseudomonas*.
